# Prognostic significance of the programmed death ligand 1 expression in clear cell renal cell carcinoma and correlation with the tumor microenvironment and hypoxia-inducible factor expression

**DOI:** 10.1186/s13000-018-0742-8

**Published:** 2018-08-25

**Authors:** Hayriye Tatli Dogan, Merve Kiran, Burak Bilgin, Aydan Kiliçarslan, Mehmet Ali Nahit Sendur, Bülent Yalçin, Arslan Ardiçoglu, Ali Fuat Atmaca, Berrak Gumuskaya

**Affiliations:** 1 Faculty of Medicine, Department of Pathology, Ankara Yıldırım Beyazıt University, Ankara, Turkey; 2 Department of Pathology, Usak University, Education and Research Hospital, Usak, Turkey; 3 Department of Oncology, Ankara Atatürk Education and Research Hospital, Ankara, Turkey; 4 Faculty of Medicine, Department of Oncology, Ankara Yıldırım Beyazıt University, Ankara, Turkey; 5 Faculty of Medicine, Department of Urology, Ankara Yıldırım Beyazıt University, Ankara, Turkey

**Keywords:** PD-L1, PD-1, HIF, Renal cell carcinoma

## Abstract

**Background:**

Clear cell renal cell carcinoma (ccRCC) is the most common renal malignancy. Hypoxia-inducible factors, HIF-1α and HIF-2α, are expressed in the majority of ccRCC. Targeting immune checkpoints with the blockade of PD-1 and its ligand PD-L1 reorganizes T-cell activity in tumor microenvironment and provides important antitumor responses. PD-L1 upregulation has been found to be hypoxia-inducible factor (HIF) dependent. Our aim is to demonstrate the association between PD-L1 and HIF expression and to reveal the role of PD-L1 in prognosis and its association with tumor microenvironment.

**Methods:**

Surgical specimens from 145 patients diagnosed with ccRCC, who had undergone radical or partial nephrectomy, were retrospectively analyzed. Immunohistochemistry on tissue microarrays (TMA) was performed to demonstrate expressions of PD-L1, HIF-1α, and HIF-2α in tumor cells and PD-1, CD4, and CD8 in lymphocytes to assess lymphocyte density in tumor microenvironment.

**Results:**

PD-L1 tumor cell expression was detected in 20/125 (13.8%) cases, which correlated with higher levels of PD-1, CD4, CD8 and HIF-2α expression. Low or high expression of HIF-1α was similar in PD-L1-positive cases. When PD-L1-positive cases were compared with negative ones, there was no significant difference in terms of prognostic factors. However, the number of WHO/ISUP grade 3–4 tumors was significantly higher in PD-L1-positive cases than in negative ones.

**Conclusion:**

PD-L1 tumor cell expression is strongly associated with increased HIF-2α expression and presence of dense lymphocytic infiltration in ccRCCs. Our findings confirm that PD-L1 positivity is associated with high ISUP nucleolar grade. The association between PD-L1, HIF, and lymphocyte density in tumor microenvironment must be clarified and especially taken into account in combination treatment.

## Background

As one of the leading therapy-resistant malignancies, renal cell carcinoma exhibits poor or no response to hormonal therapy, radiotherapy, and chemotherapy [1]. Clear cell renal cell carcinoma (ccRCC) comprises about 3% of adult cancer cases and is an aggressive tumor characterized in most cases by the inactivation of the tumor -suppressor gene *VHL*. The Von Hippel–Lindau protein (pVHL) regulates the hypoxia-inducible factor (HIF), and the loss of the pVHL function causes constitutive stabilization of HIF-1α and HIF-2α, resulting in the induction of HIF-transcriptional targets [2]. Reportedly, HIF activation is an important oncogenic driving force in ccRCC. In fact, HIF-1α and HIF-2α play a crucial role in the tumor growth and tumor progression, respectively [3, 4]. Furthermore, HIF activates genes, which encode proteins associated with angiogenesis and cell -cycle regulation.

The pVHL–HIF–VEGF pathway plays an essential role in angiogenesis and is targeted by anti-angiogenic therapy. Targeted therapies, including vascular endothelial growth factor and the mTOR pathway inhibitors, demonstrate a prolonged progression-free and overall survival rate; no durable response has yet been achieved. In addition, targeted therapies affecting the downstream of the pVHL–HIF pathway or mTOR are characterized by toxic side effects. Recently, targeting immune checkpoints has been reported as a novel approach for cancer treatment [5].

The blockage of programmed cell death 1 (PD-1) and its interference with its ligand, programmed death ligand 1 (PD-L1) reorganizes the T-cell activity in the tumor microenvironment and facilitates critical antitumor responses. PD-1 is a cell-surface protein of 288 amino acids, which is an immune inhibitor, and is expressed because of the immunological activation of PD-1^+^, CD4^+^, CD8^+^ T cells, NK cells, B cells, and monocytes [6]. Previously, two PD-1 ligands (PD-L1, also called B7-H1, and PD-L2, also known as B7-DC) have been described. While PD-L1 is expressed in several cells, including resting T cells, B cells, macrophages, dendritic cells (DCs), and vascular endothelial cells, PD-L2 is expressed only in macrophages and DCs [7]. Downregulation of T-cell response is because of the interaction between PD1 and PD-L1–PD-L2 ligand expressed by tumor cells. The inhibition of the PD-1–PD-L1 axis increases the T-cell proliferation and cytotoxicity, indicating a promising mechanism to stimulate the antitumor activity of the immune system [8]. In addition, PD-L1, which is expressed in tumor cells, contributes to metastasis and progression, repressing any T-cell–mediated immune response against cancer. Although the PD-L1 expression is a potential predictive biomarker, it also correlates with the worst clinical course in tumors, including renal cell cancers [9–14]. As a result, the need of developing novel therapies for the blockage of PD-1 has been recognized in the recent research [12].

Although the *VHL* function loss is a critical factor in the RCC progression, some recent studies have identified the correlation between the *VHL* mutation and the immune checkpoint molecules. However, only a few studies have established a strong correlation between the *VHL* mutation and PD-L1 expression. In addition, studies with cell culture experiments have reported that HIF-2α stabilization, induced by the *VHL* mutation, together with impaired pVHL increased the PD-L1 expression, suggesting that PD-L1 is a HIF-2α target, which is upregulated in pVHL-deficient ccRCC [5, 8]. Furthermore, other studies have emphasized that an increase in the HIF (HIF-1α or HIF-2α) expression is associated with poor prognostic factors and that it might be a potential biomarker [15, 16]. Notably, the combination of PD-L1 targeting drugs with HIF-inhibiting agents could be an additional option for the treatment of ccRCC.

As HIF-α is essential in tumorigenesis and is one of the primary aims of treatment, especially when the correlation between HIF-2α and PD-L1 has recently been demonstrated in some cell culture studies, this study aims to investigate the expression condition of PD-L1, HIF-1α and HIF-2α proteins in human renal tumor tissues using the immunohistochemical method and assess whether the HIF-2α or HIF-1α increases in PD-L1-positive cases. Furthermore, this study aims to determine the correlation between the PD-L1 tumor cell expression and clinicopathological prognostic factors, lymphocyte density displaying PD-1, CD4, and CD8 expression in the tumor microenvironment in ccRCC series.

## Methods

### Patients

In this study, surgical specimens obtained from 145 patients (males, 93 [64.9%]; females, 52 [35.1%]; mean age: 57.8 ± 11.1 [range: 31–86] years) with ccRCC who underwent radical or partial nephrectomy between 2007 and 2014 at our institution were retrospectively assessed. While 102 (70.3%) samples were obtained from patients who underwent radical nephrectomy, 43 (29.7%) were obtained from those who underwent partial nephrectomy. We analyzed clinical and pathological characteristics, including age, gender, tumor size, WHO/ISUP nucleolar grade [17], coagulative tumor necrosis, microvascular invasion, renal pelvis, adrenal, ureter, renal vein invasion, distant metastasis, and overall survival. Clinicopathological characteristics are summarized in Table 1. This study protocol was approved by the Ethical Committee of Ankara Yıldırım Beyazıt University.

**Table 1:** Summary of the clinical and histopathological characteristics of 145 patients with ccRCC

Variables	Number of patients
Sex	
M	93 (64.9%)
F	52 (35.1%)
Age (years)	57.8 (31–86)
Tumor size (cm)	5.76 (1.5–18)
Nucleolar grade	
1	17 (11.7%)
2	57 (39.3%)
3	43 (29.7%)
4	28 (19.3%)
Lymph node metastases	
no	136 (93.8%)
yes	9 (6.2%)
Distant metastases	
no	115 (79.3%)
yes	30 (20.7%)
Sarcomatoid component	
no	137 (94.5%)
yes	8 (5.5%)

### Tissue microarray and immunohistochemistry staining procedure

Tissue microarray (TMA) blocks using a precision mechanical system (Quick Ray, Manual Tissue Microarrayer, UNITMA, Korea) were constructed. From each patient with ccRCC, two tissue samples (diameter, 0.3 cm each) corresponding to the previously demarcated and most representative areas of respective hematoxylin–eosin-stained slides were removed. One of the core biopsies was sampled from the center and the other was sampled from the advancing edge of the tumor. Afterwards, these samples were transferred to a recipient paraffin block at 3-mm intervals. Finally, TMA blocks were cut into 4-μm histological sections and used for immunohistochemistry.

An immunohistochemical analysis for the expression of PD-L1 (SP263; 1:100 dilution; Ventana), PD-1 (NAT 105; 1:250 dilution; Cell Marque), CD4 (SP351; 1:100 dilution; Ventana), CD8 (SP57; 1:100 dilution; Ventana), HIF-1α (H1alpha67; 1:50 dilution; Novus), and HIF-2α (ep190; 1:100 dilution; GeneTex) were performed. All sections were stained with primary antibodies on Ventana GX benchmark equipment with standard antigen retrieval (CC1 buffer; pH 8.0; Ventana). An ultraView Universal DAB Detection Kit (Ventana) per the manufacturer’s instructions were used. In addition, counterstaining was performed as part of the automated staining protocol using hematoxylin. After staining, the slides were washed, dehydrated in graded alcohol and xylene, mounted, and coverslipped. To verify antibody specificity, tonsil tissue was used as a positive control.

The PD-L1 expression in tumor cells in TMAs was quantified. The membranous PD-L1 expression with or without cytoplasmic staining in ≥5% tumor cells was considered positive. In addition, the tumor microenvironment categorization was performed depending on the density of CD4-, CD8-, and PD-1-expressing lymphocytes in the tumor microenvironment. The expression of CD4, CD8, and PD-1 in lymphocytes was scored as 1 point (0–4 cells per high-power field, ×400), 2 points (5–8 cells), 3 points (9–12 cells), and 4 points (≥13 cells) [18].

For HIF-1α and HIF-2α, the staining was scored according to the intensity and percentage of tumor cells. The intensity was scored 0 (no staining), 1 (weak), 2 (moderate), and 3 (strong staining). The percentage staining was scored as 0 (no staining), 1 (1–10%), 2 (11–50%), 3 (51–90%), and 4 (91–100%). In addition, the histoscore was calculated for analysis using the product of these two scores, giving a resultant score from 0 to 12. The final histoscore used for the data analysis was the mean score from the two tissue cores from each ccRCC tumor block. Finally, the median histoscore was used to evaluate the cutoff scores for “high” or “low” staining for each protein [15].

### Statistical analysis

All patient data were entered to statistical analysis software. Using the Shapiro–Wilk test, the normal distribution of continuous variables was graphically evaluated. In this study, all continuous variables were skewed. Categorical variables (gender, PD-L1 condition, LVI, etc.) to determine identical statistics and the median values to present the number and percentage measurement variables were used. Using the Mann–Whitney *U*-test, differences between continuous variables in accordance with the PD-L1 condition were evaluated. In addition, crosstabs and calculated *χ*^2^ values to determine differences between categorical variables in accordance with the PD-L1 condition were created. Next, the time to recurrence was calculated using the Kaplan–Meier method, and the differences between the curves were assessed using the log-rank test. MS-Excel 2010 and the IBM SPSS Statistics 21.0 (released in 2012; IBM SPSS Statistics for Windows, Version 21.0; IBM Corp., Armonk, NY) were used for statistical analyses and calculations. Furthermore, *p* < 0.05 was considered statistically significant.

## Results

**Fig. 1:**
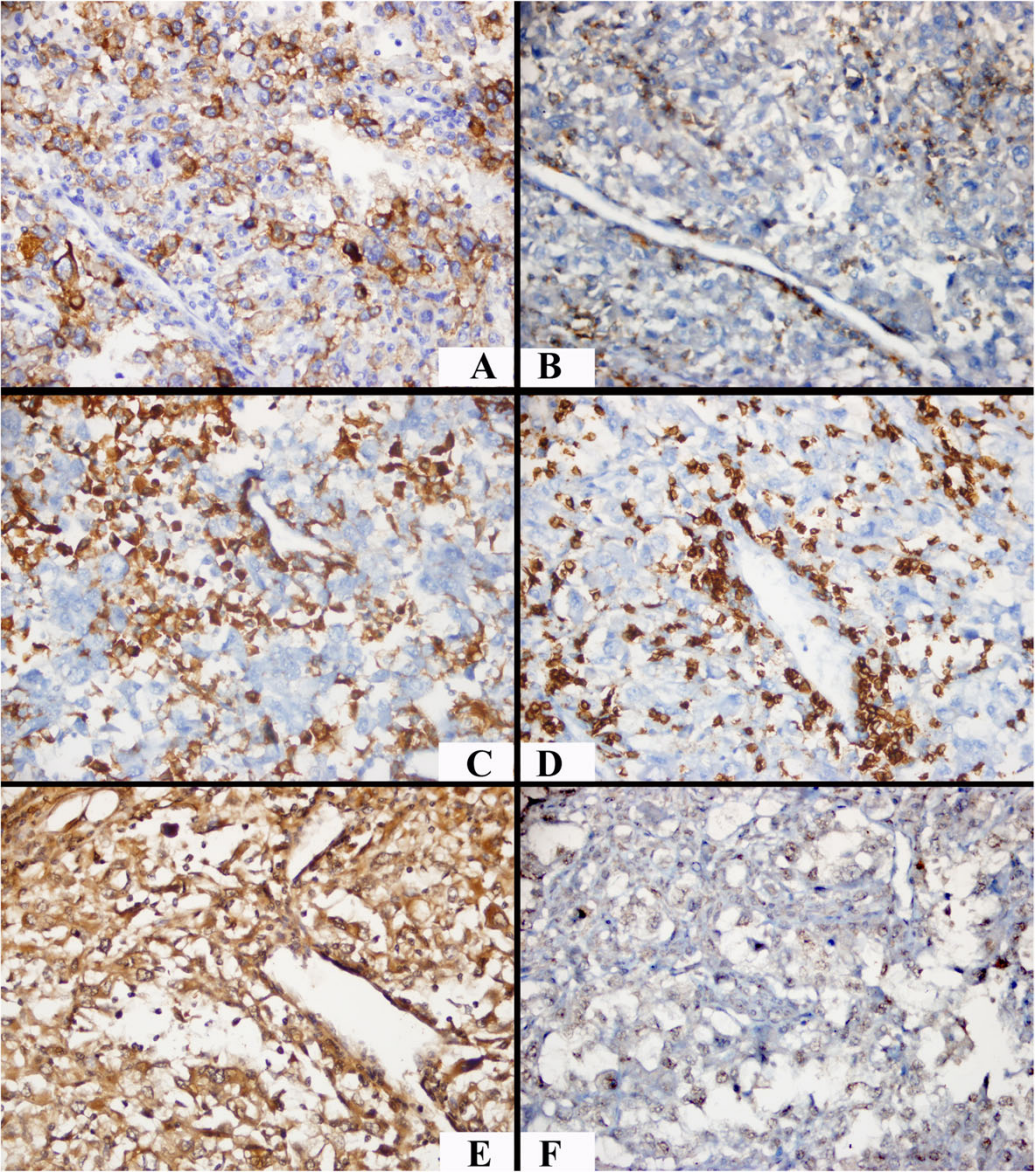
A PD-L1-positive case with an increased lymphocyte density in the microenvironment and high HIF-2α, low HIF-1α expression (× 400). **a** PD-L1 positivity with prominent membranous and scarce cytoplasmic staining. **b** PD-1-positive dense lymphocytic infiltration. **c** CD4-positive dense lymphocytic infiltration. **d** CD8-positive dense lymphocytic infiltration. **e** High-score HIF-2α expression. **f** Low-score HIF-1α expression

In this study, the PD-L1 tumor cell expression rates were 0–90%. While samples with a PD-L1 staining rate of ≥5% were considered positive, those with < 5% rate were considered negative. Based on the PD-L1 results, 125 (86.2%) samples were negative, whereas only 20 (13.8%) were positive. Likewise, samples with scores of 0–2 for PD1, CD4, and CD8 were encoded as “none or mild,” whereas scores of 3–4 were encoded as “dense” lymphocytic infiltration in the tumor microenvironment. When PD-L1 exhibited positive results, the rate of dense lymphocytic infiltration staining with PD-1, CD4, and CD8 was significantly high (*P* < 0.05; Table 2; Fig. 1a–d).

**Table 2:** PD-1, CD4, CD8, HIF-1α, and HIF-2α expression according to PD-L1 results

		PD-L1 Staining		Test Statistics (χ2; P)
		Negative n (%)	Positive n (%)	
PD-1	Mild	92 (73.6)	6 (30.0)	14.961; 0.000
	Dense	33 (26.4)	14 (70.0)	
CD4	Mild	64 (51.2)	2 (10.0)	11.801; 0.001
	Dense	61 (48.8)	18 (90.0)	
CD8	Mild	66 (52.8)	2 (10.0)	12.682; 0.000
	Dense	59 (47.2)	18 (90.0)	
HIF-1α	Low	55 (44.0)	11 (55.0)	0.841; 0.359
	High	70 (56.0)	9 (45.0)	
HIF-2α	Low	57 (45.6)	4 (20.0)	4.636; 0.031
	High	68 (54.4)	16 (80.0)

**Fig. 2:**
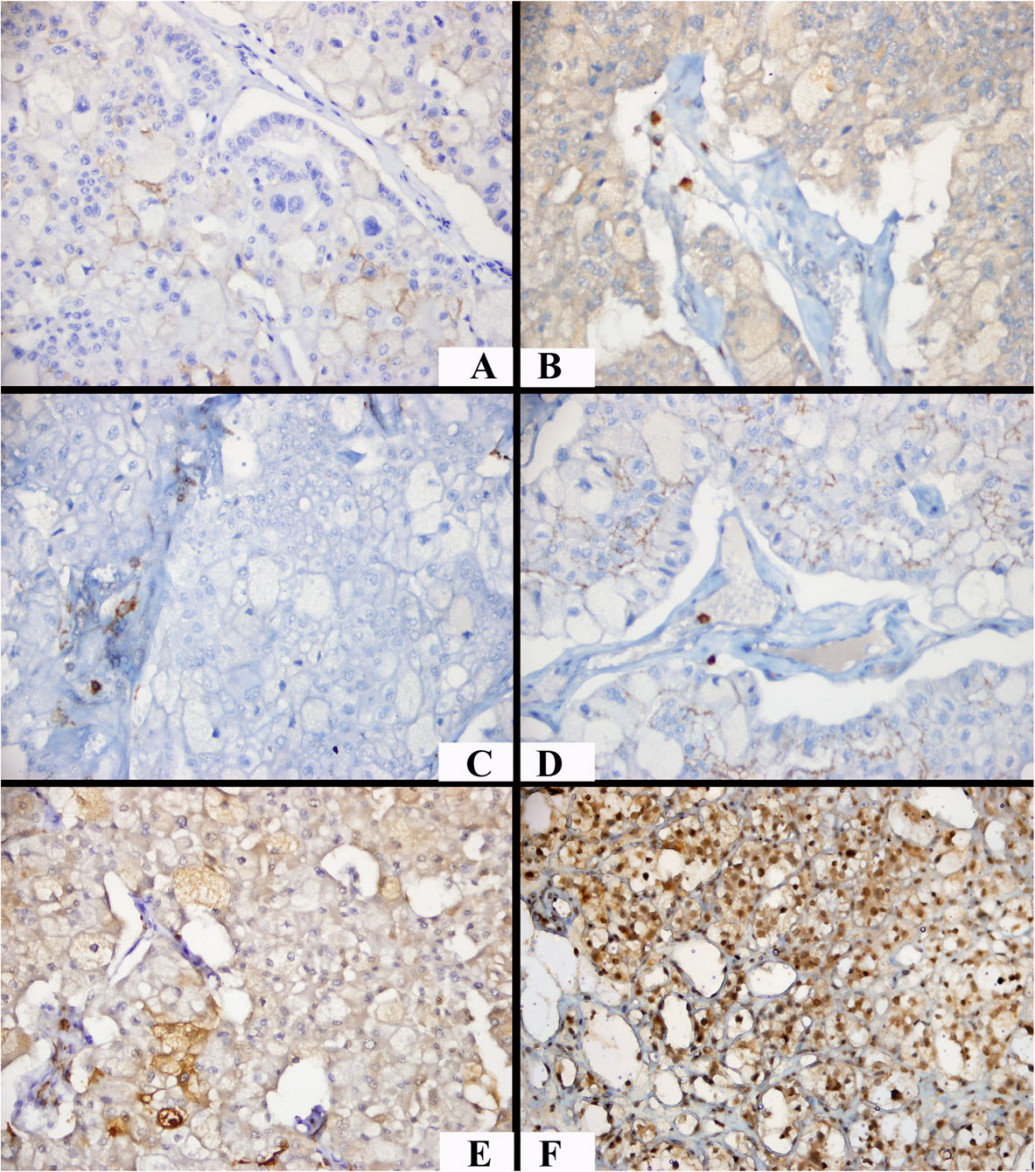
A case exhibiting PD-L1-expression in a few cells with a mild lymphocyte density in the microenvironment, low HIF-2α expression, and high HIF-1α expression (× 400). **a** Scarce incomplete membranous PD-L1 staining. **b** PD-1-positive mild lymphocytic infiltration, **c** CD4-positive mild lymphocytic infiltration. **d** CD8-positive mild lymphocytic infiltration, **e** Low-score HIF-2α expression. **f** High-score HIF-1α expression

**Fig. 3:**
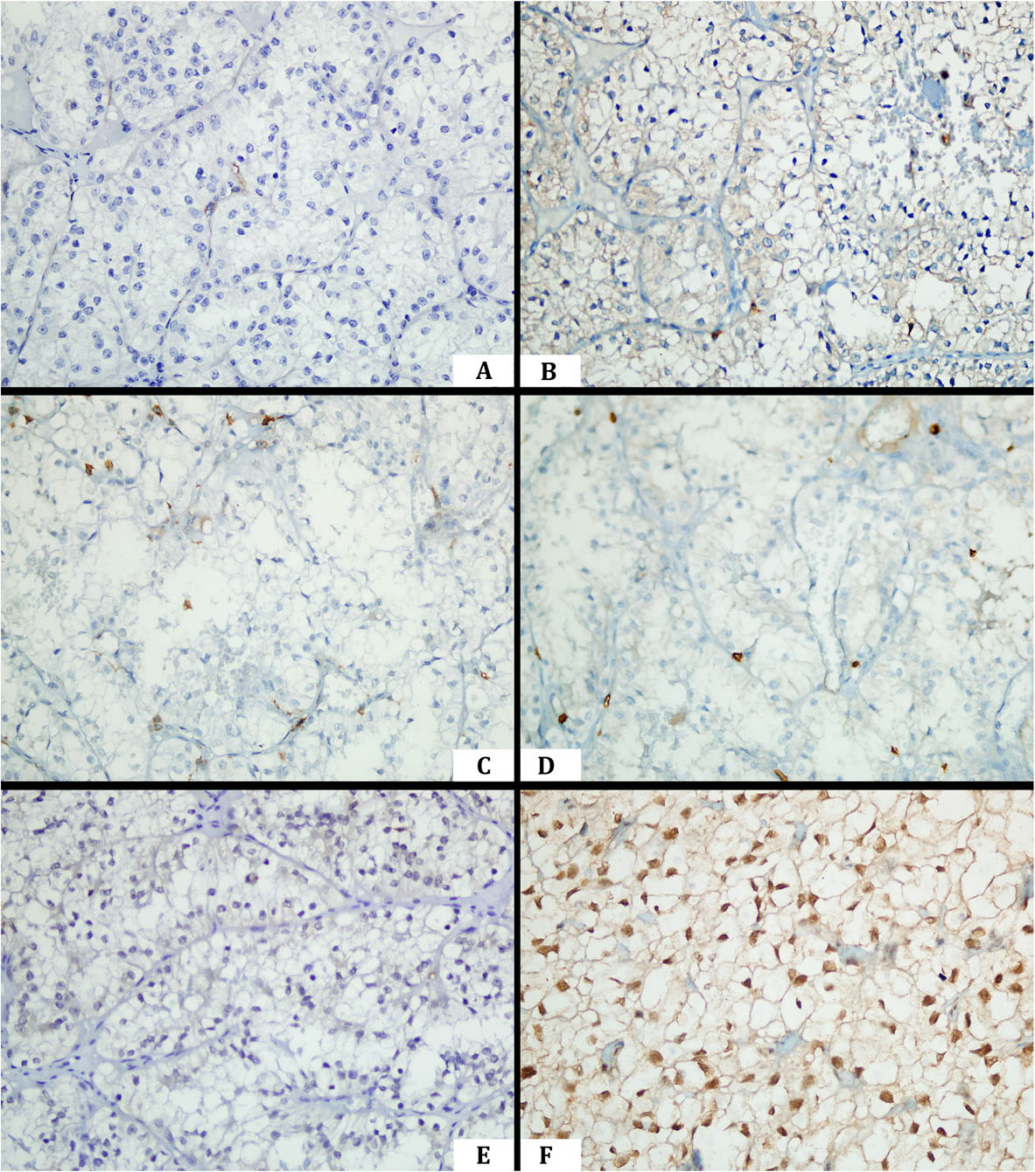
A PD-L1 negative case with a mild lymphocyte density, low HIF-2α expression, and high HIF-1α expression (× 400). **a** No PD-L1 staining. **b** PD-1-positive mild lymphocytic infiltration, **c** CD4-positive mild lymphocytic infiltration. **d** CD8-positive mild lymphocytic infiltration, **e** Negative HIF-2α expression. **f** High-score HIF-1α expression

While HIF-1α was expressed in the nuclei of ccRCC cells, HIF-2α was expressed in the cytoplasm. Samples with HIF-1α scores < 3 were classified as “low,” whereas those with ≥3 scores were classified as “high.” In contrast, the classification of “low” was used for HIF-2α scores < 6, and “high” was used for ≥6. When PDL-1 revealed positive results, the low or high occurrence of HIF-1α scores was similar (χ2 = 0.841; *p* = 0.359). In addition, the rate of high HIF-2α expression scores was significantly higher in PD-L1-positive cases (χ2 = 4.636; *p* = 0.031; Table 2; Fig. 1e and f). The majority of the cases with scarce PD-L1 expression or no PD-L1 expression showed mild lymphocytic infiltration and low HIF-2 α expression (Figs. 2 and 3).

The results of the PD-L1 tumor cell expression in accordance with the prognostic factors were assessed. When PD-L1-positive cases were compared with the negative ones, no significant difference was observed in terms of most of the prognostic factors (*p* > 0.05). As an exception the rate of WHO/ISUP grade 3–4 tumors was considerably higher in PD-L1-positive cases than that in negative ones (Table 3). Although the mean tumor size was 7.6 cm in PD-L1-positive cases, it was 5.4 cm in PD-L1-negative ones; however, this difference was not statistically significant (*p* = 0.11). When cases with PD-1, CD4, CD8 expression scores of 0–2 were compared with those with scores of 3–4, there was no significant difference in terms of the same prognostic factors (*p* > 0.05). In our study, only 28 cases were grade 4. Compared with the rest of the cases, grade 4 cases had more dense PD-1 positive lymphocytes in their tumor microenvironments (*p* = 0.027). But there was no statistically significant difference in terms of PDL-1, CD4, CD8, and HIF expressions in grade 4 tumors.

**Table 3:** Summary of clinicopathologic prognostic factors according to the PD-L1 status

		PD-L1 Staining		Test Statistics (χ2; P)
		Negative n (%)	Positive n (%)	
Renal Pelvis Invasion	No	115 (92.0)	18 (90.0)	0.091; 0.763
	Yes	10 (8.0)	2 (10.0)	
Adrenal gland direct Invasion	No	123 (98.4)	20 (100)	0.324; 0.569
	Yes	2 (1.6)	0 (0)	
Adrenal Gland Metastasis	No	123 (98.4)	19 (95)	0.754; 0.384
	Yes	2 (1.6)	1 (5)	
Sarcomatoid Component	No	119 (95.2)	18 (90)	0.894; 0.344
	Yes	6 (4.8)	2 (10)	
Necrosis	No	80 (64.0)	13 (65)	0.007; 0.931
	Yes	45 (36.0)	7 (35)	
Lymphovascular Invasion	No	109 (87.2)	16 (80)	0.752; 0.386
	Yes	16 (12.8)	4 (20)	
Ureter Invasion	No	123 (98.4)	20 (100)	0.324; 0.569
	Yes	2 (1.6)	0 (0)	
Renal Vein Invasion	No	117 (93.6)	18 (90)	0.348; 0.555
	Yes	8 (6.4)	2 (10)	
Distant Metastasis	No	100 (80.0)	15 (75)	0.263; 0.608
	Yes	25 (20.0)	5 (25)	
Lymph Node Metastasis	No	117 (93.6)	19 (95.0)	0.058; 0.810
	Yes	8 (6.5)	1 (5.0)	
WHO/ISUP grade	1–2	68 (54.4)	6 (30)	4.108; 0.043
	3–4	57 (45.6)	14 (70)	

In this study, the median length of follow-up was 48 months, and the mean survival was 90.06 months for PD-L1-positive cases and 94.9 months for PD-L1-negative cases; this difference was not statistically significant (*p* = 0.359). There was no significant difference among the cases with PD-1, CD4, CD8 expression scores of 0–2 and 3–4 in terms of overall survival (*p* < 0.05).

## Discussion

Studies have reported that the PD-L1 expression is indicative of a response to new PD-1/PD-L1 inhibitors in several tumors, including RCC [13, 19]. In addition, the clinicopathological and prognostic value of PD-L1 has been investigated in some recent studies and meta-analyses, in which several clinicopathological prognostic factors of renal tumors, such as the WHO/ISUP grade, presence of necrosis, tumor size, TNM stage have been compared with the PD-L1 expression, and a considerable correlation between some of these factors has been determined. In addition, most studies have suggested that patients with intratumoral high PD-L1 expression exhibited aggressive tumors and have increased risk of death from RCC [1, 12, 19–21].

One of the most extensive meta-analysis investigating 1863 patients from 10 studies reported that the PD-L1 expression is correlated with poor overall survival in ccRCC and non-ccRCC. In addition, the PD-L1 expression was highly correlated with the primary tumor stage, regional lymph node metastases, distant metastases, nuclear grade, and tumor necrosis. In this meta-analysis, the PD-L1 expression is determined in 414 cases (29.5%) in the ccRCC population [22]. However, we demonstrated that PD-L1 tumor cell expression was 13.8% in ccRCC cases. The cutoff value used to evaluate PD-L1 positivity were ≥ 5% in seven out of the 10 studies in the content of this meta-analysis and therefore similar to our study. In fact, the reported rate of the PD-L1 positivity was between 15 and 66%, which is indicative of high variability [19, 20, 23].

Compared with other studies, the lower rate of the PD-L1 tumor cell expression in this study and the reported rate of high variability in previous studies could be attributed to the use of different anti-PD-L1 antibodies, different cutoff values, and tumor heterogeneity. Of note, ccRCC is a neoplasm characterized by intratumoral heterogeneity [24]. The use of the TMA to indicate the PD-L1 expression in this study could be a pitfall because of tumor heterogeneity. In addition, the PD-L1 expression might vary within the primary tumor or in the primary tumor versus metastases, which might limit the predictive value of this biomarker. Callea et al. reported discordant staining of PD-L1 between primary tumors and corresponding metastases in a high proportion of cases [25]. In this study, PD-L1-positivity correlated with high WHO/ISUP nucleolar grade. Notably, studies investigating the prognostic significance of PD-L1 are comparatively fewer in RCC, and the results are not entirely consistent [26, 27].

Furthermore, ccRCCs harboring the PD-L1 expression significantly correlated with PD-1-, CD4-, and CD8-positive dense inflammatory response. In our study, it was demonstrated that, mononuclear cells that infiltrate the RCC tumor microenvironment express PD-1. Thompson et al. reported that patients with PD-1-positive immune cells tended to harbor more B7-H1^+^ tumor cells and larger and higher-grade tumors. In addition, the cancer-specific death rate was considerably higher in patients with PD-1-positive immune cells compared with PD-1-negative ones [14]. Perhaps, interactions between immune cell PD-1 and PD-L1 might promote the cancer progression by contributing to immune dysfunction in patients with ccRCC. Reportedly, immune-regulated tumors exhibited aggressive histological features, a high risk of disease progression, and a CD8^+^PD-1^+^ and CD4^+^ phenotypic signature [28]. Besides PD-L1, the identification of the lymphocyte density in tumor microenvironment as a prognostic biomarker could facilitate detecting patients who could benefit from the checkpoint blockade.

Hypoxia is crucial for the PD-L1 up regulation. PD-L1 is the B7 family member of immune-regulatory ligands and could be stimulated by hypoxia [29]. This situation has led researchers to investigate whether the PD-L1 expression in ccRCC is due to the deregulation of the pVHL–HIF axis [5]. Loss of the pVHL function causes HIF stabilization acting as a pseudo-hypoxic condition in most ccRCCs. Ruf et al. reported that the PD-L1 up regulation in ccRCC cell lines was determined to be HIF-dependent and driven predominantly by the HIF-2α subunit, which accumulated in ccRCC because of VHL protein inactivation. In contrast, the knockdown of HIF-2α, but not of HIF-1α, decreased PD-L1 protein levels in ccRCC cell lines [5]. Our study showed a strong association of increased levels of the HIF-2α expression in PD-L1-positive ccRCC tumor samples by immunohistochemistry, demonstrating the correlation between the increased PD-L1 and HIF expression, suggesting that the regulation of PD-L1 might depend on the HIF expression [5, 8]. Few studies have demonstrated the direct binding of HIF-1α to a transcriptionally active region in the PD-L1 proximal promoter, indicating PD-L1 as a potential target for HIF-1α [30]. In our study, there was no association between PD-L1 expression and HIF 1α. VHL-defective RCC cells tend to express more HIF-2α than HIF-1α protein [4] as the ccRCC progress the HIF-1α/ HIF-2α balance shifts toward HIF-2α.

Kammerer et al. have found PD-L1 expression to be associated in particular with non-inactivated wild-type *VHL* tumors [11]. Beuselinck et al. have reported PD-L1 expression and fewer *VHL* gene mutations in ccRCC4 tumors [31].

These reports suggest that alternative oncogenic pathways in ccRCC may lead to PD-L1 overexpression despite HIF degradation due to the presence of an activated VHL protein. Tumors without the inactivation of VHL could use alternative pathways, such as the MAP kinase and PI3K- AKT–mTOR pathways involved in ccRCC oncogenesis [32].

The emergence of the molecular basis of hypoxia and angiogenesis has enabled the development of treatments toward HIF-related pathways, like phosphatidylinositol 3-kinase–AKT–mTOR, RAS–RAF–MAP, and VEGF signal pathways. Although these agents result in positive recovery without progression, clinical resistance exists. Recently, treatments toward HIF with agents such as histone deacetylase inhibitors and HIF-2α antagonists have been reported [33, 34]. Presumably, the revelation that the correlation between the HIF pathway and the immune response is associated with PD-L1 could be useful for the enlightenment of tumor biology. Moreover, there could be more positive results regarding HIF-2α antagonists, which are yet to be added to immunotherapy.

This study has some limitations. One major limitation of studies reporting PD-L1 staining to date, including this study, is the use of various antibodies and variability in staining methodologies. However, antibody used in our study has recently been approved by FDA. Thus, standardization of staining procedures and scoring methods is warranted before PD-L1 and PD-1 could be widely used as predictive biomarkers [35]. Although recent studies suggest evaluating PD-L1 status in both components (either immune cells or tumor cells), in this study, PD-L1 expression of tumor infiltrating lymphocytes was not assessed [28, 36]. Instead, PD-L1 expression in tumor cells and its interaction with PD-1, CD4, CD8-positive lymphocytes was our center of interest. The use of TMA sections instead of whole sections is another restriction in our study because of tumor heterogeneity. Another limitation of this study was the inability to assess the *VHL* mutation in patients. However, the HIF expression status reflected the deficient VHL–HIF axis or hypoxia. Since VHL pathway is unperturbed in clear cell papillary RCC, these cases could act as negative controls. However, clear cell papillary RCC cases have not been tested in this study.

Hypoxia considerably increases the PD-L1 expression on macrophages, myeloid-derived suppressor cells, DCs, and tumor cells. HIF-induced tumor cell PD-L1 expression contributes to cancer immune evasion. The blockade of PD-L1 under hypoxia enhances T-cell activation [30]. In addition, blocking the HIF accumulation prevented the hypoxia induced PD-L1 expression and caused resistance to tumor cell lysis mediated by cytotoxic T cells [37]. This suggests that combining therapies blocking PD-1/PD-L1 with agents targeting HIF could further improve the tumor cell clearance by breaking the immune evasion in RCC [5].

## Conclusion

In our study, PD-L1 tumor cell expression was 13.8% of the ccRCC cases with SP263 clone and it was correlated with higher levels of PD-1, CD4, and CD8. Moreover, PD-L1 expression was associated with high HIF-2α expression indicating the possibility of PD-L1 regulation by HIF-2α. However, there was no correlation between PD-L1 tumor cell expression and HIF-1α scores. In PD-L1-positive cases, there was no significant difference in terms of the most of the prognostic factors except for the high WHO/ISUP grade. A patient presenting with a high-grade tumor with dense lymphocytic infiltration can be candidate for PD-L1-targeted therapy. These morphological characteristics may be premonitory for further immunohistochemical tests. There are several suggested mechanisms regulating PD-L1 expression, such as VHL, hypoxia, or other alternative pathways. This study highlights the necessity of determining biological characteristics of tumors to elucidate the specific correlation between tumor-driving pathways and the immune system and precisely assess the tumor microenvironment to determine predictive biomarkers for the identification of tailored treatment. The interactions between potential biomarkers must be determined accurately, especially when considering the combination treatment.

## Abbreviations

ccRCC: Clear cell renal cell carcinoma
DCs: Dendritic cells
HIF: Hypoxia-inducible factor
PD-1: Programmed death 1
PD-L1: Programmed death ligand 1
PD-L2: Programmed death ligand 2
pVHL: von Hippel–Lindau protein
TMA: Tissue microarray
